# Nutritional Approaches to Modulate Cardiovascular Disease Risk in Systemic Lupus Erythematosus: A Literature Review

**DOI:** 10.3390/nu15041036

**Published:** 2023-02-19

**Authors:** Karen Pesqueda-Cendejas, Melissa Rivera-Escoto, Mónica R. Meza-Meza, Bertha Campos-López, Isela Parra-Rojas, Margarita Montoya-Buelna, Ulises De la Cruz-Mosso

**Affiliations:** 1 Red de Inmunonutrición y Genómica Nutricional en las Enfermedades Autoinmunes, Centro Universitario de Ciencias de la Salud, Universidad de Guadalajara, Guadalajara 44340, Jalisco, Mexico; 2 Instituto de Neurociencias Traslacionales, Departamento de Neurociencias, Centro Universitario de Ciencias de la Salud, Universidad de Guadalajara, Guadalajara 44340, Jalisco, Mexico; 3 Programa de Doctorado en Ciencias de la Nutrición Traslacional, Centro Universitario de Ciencias de la Salud, Universidad de Guadalajara, Guadalajara 44340, Jalisco, Mexico; 4 Programa de Doctorado en Ciencias Biomédicas Orientación Inmunología, Centro Universitario de Ciencias de la Salud, Universidad de Guadalajara, Guadalajara 44340, Jalisco, Mexico; 5 Programa de Doctorado en Ciencias en Biología Molecular en Medicina, Centro Universitario de Ciencias de la Salud, Universidad de Guadalajara, Guadalajara 44340, Jalisco, Mexico; 6 Laboratorio de Investigación en Obesidad y Diabetes, Facultad de Ciencias Químico-Biológicas, Universidad Autónoma de Guerrero, Chilpancingo de los Bravo 39087, Guerrero, Mexico; 7 Laboratorio de Inmunología, Departamento de Fisiología, Centro Universitario de Ciencias de la Salud, Universidad de Guadalajara, Guadalajara 44340, Jalisco, Mexico

**Keywords:** systemic lupus erythematosus, cardiovascular disease risk, dyslipidemia, hypertension, hyperhomocysteinemia, C-reactive protein

## Abstract

Systemic lupus erythematosus (SLE) is a chronic pathology characterized by a bimodal mortality pattern attributed to clinical disease activity and cardiovascular disease (CVD). A complex interaction between traditional CVD risk factors such as obesity, dyslipidemia, smoking, insulin resistance, metabolic syndrome, and hypertension, as well as the presence of non-traditional CVD risk factors such as hyperhomocysteinemia, pro-inflammatory cytokines, and C-reactive protein levels, has been suggested as a cause of the high prevalence of CVD in SLE patients. On the other hand, environmental factors, such as nutritional status, could influence the disease’s prognosis; several nutrients have immunomodulators, antioxidants, and anti-cardiometabolic risk properties which could reduce SLE severity and organ damage by decreasing the development of traditional and non-traditional CVD risk factors. Therefore, this critical literature review discusses the therapeutic potential of nutritional approaches that could modulate the development of the main comorbidities related to CVD risk in SLE patients.

## 1. Introduction

Systemic lupus erythematosus (SLE) is the prototype autoimmune inflammatory disease characterized by the production of autoantibodies against self-antigens, such as deoxyribonucleic acid (DNA), proteins, and nucleosomes [1]. Notably, cardiovascular disease (CVD) is one of the most important causes of death described in SLE patients [2,3]. Mortality in SLE is characterized by the involvement of traditional and non-traditional CVD risk factors, such as obesity, dyslipidemia, atherosclerosis, and high serum levels of pro-inflammatory mediators. The factors mentioned above could modulate the severity of clinical disease activity, organ damage, and chronicity in SLE [2]. Concerning this, SLE patients have a five- to sixfold higher CVD risk and a fiftyfold higher risk for acute myocardial infarction than the general population [3]. Moreover, mortality in SLE patients presents a bimodal pattern, with an initial peak owing to clinical disease activity and a late peak attributable to the development of atherosclerosis and CVD. The risk of developing CVD and subclinical atherosclerosis is increased in SLE patients; this increase is partly explained by traditional CVD risk factors, such as smoking, hypertension, and dyslipidemia [4]; nevertheless, the central axes of CVD risk in SLE are dyslipidemia, high serum levels of pro-inflammatory mediators, autoantibodies, and obesity [5]. Epidemiological studies have shown that dyslipidemia could be diagnosed in 50–85% of children and adolescents with SLE. It indicates a high incidence of atherosclerosis and a worse disease prognosis [6]. Therefore, SLE has historically been considered an independent CVD risk factor [7].

Dyslipidemia and obesity in SLE patients could also be influenced by the pharmacotherapy administered, such as prednisone administration, which has been correlated with high levels of total cholesterol triglycerides (TG), low-density lipoprotein-cholesterol (LDL-C), glucose intolerance; lower levels of high-density lipoprotein-cholesterol (HDL-C); altered arterial blood pressure; and high body mass index (BMI) [8,9]. Obesity is the main traditional CVD risk comorbidity that triggers the development of glucose intolerance, insulin resistance (IR), atherogenic dyslipidemia, and pro-inflammatory status—conditions associated with high clinical disease activity in SLE [5,10,11,12]. Obesity in SLE increases the expression of interleukin 23 (IL-23), tumor necrosis factor alpha (TNF-α), IL-6, and C-reactive protein (CRP) [12]. This sustained inflammatory status modulated by obesity could play a central role in clinical disease activity through a positive feedback loop with pro-inflammatory mediators, such as CRP [4,13]. A high CRP concentration in an obese SLE patient linearly increases the risk of CVD, and CRP is an independent predictor of SLE, possibly attributed to its pro-inflammatory effect in obesity [14].

In addition, an imbalance of nutritional status and inadequate nutrient intake have been implicated as risk factors for exacerbating SLE’s clinical manifestations [12,15]. Dietary habits directly correlate with BMI, and a dietetic restriction of macronutrients and calories has been described to be beneficial in reducing disease manifestations in SLE-prone mice [16]. Previous studies have reported that usually, SLE patients have a high risk of malnutrition characterized by micronutrient deficiencies, low bone mineral density, hyperhomocysteinemia, and altered immune response, compared with the general population [5,17]. These events are related to a high CVD risk. In this way, several nutrients have been described that could modulate the inflammatory response through their immunomodulatory properties. Their intake could contribute to an adequate nutritional and anti-inflammatory status, modifying the prognosis and development of comorbidities related to CVD in SLE patients [15,17].

Previous studies have shown that nutritional status and diet represent risk factors that can be modified with nutritional intervention and prevent the development of the main CVD comorbidities [18,19]. However, more evidence is needed to design a cardioprotective diet for SLE patients. Therefore, based on this knowledge, this critical literature review aims to provide historical and recent evidence to discuss the therapeutic potentials of nutritional approaches that could modulate the development of the main comorbidities related to CVD in SLE.

## 2. Materials and Methods

**Figure 1:**
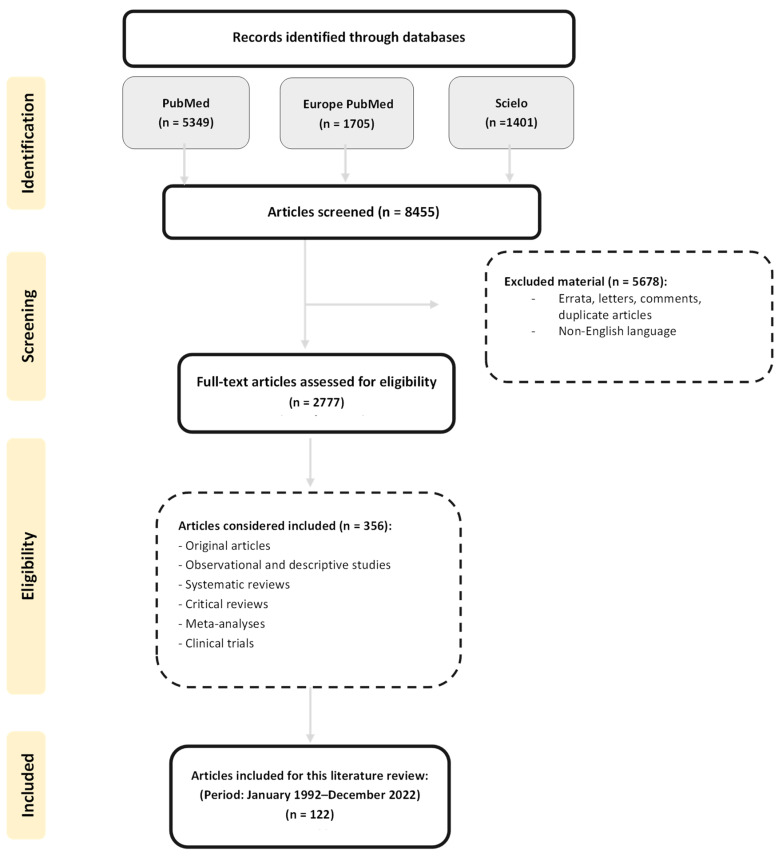
Methodological diagram of the literature search and selection process.

A compressive literature search was performed in the following databases and search engines: PubMed, Europe PubMed Central, and Scielo, considering the period January 1992 to December 2022. The following keywords were used to obtain information on the topics and subtopics: “systemic lupus erythematosus” AND “cardiovascular disease” OR “cardiovascular risk” in combination with the terms: “nutritional status”, “nutrients”, “polyunsaturated fatty acids”, “vitamin A”, “vitamin C”, “vitamin E”, “vitamin B”, “vitamin D”, “selenium”, “polyunsaturated fatty acids”, “vitamin C”, “vitamin E supplementation”, “B vitamins supplementation”, “coenzyme Q10 supplementation”, “probiotics supplementation”, “dietary fiber”, “vitamin A supplementation” and “selenium supplementation”. Likewise, the methodology and quality of the articles were carefully reviewed, and a complementary bibliography of each selected article was used to find more relevant information. Only English articles were considered. Errata, letters, comments, editorials, and duplicate articles were excluded. Selected articles for the literature review included: original articles; observational and descriptive studies; systematic reviews; critical reviews; meta-analyses; and clinical trials in human and murine models. The article selection methodology is illustrated in Figure 1.

## 3. Cardiovascular Disease Risk Factors in SLE

**Figure 2:**
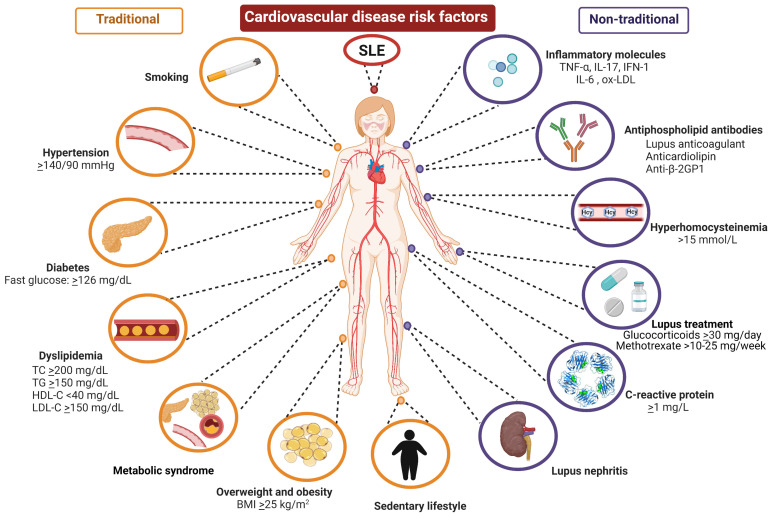
Traditional and non-traditional cardiovascular disease risk factors in SLE patients. SLE patients have a high prevalence of traditional risk factors for CVD, which are also common in the general population. However, SLE patients have CVD risk factors related to SLE pathophysiology and SLE pharmacotherapy, known as non-traditional CVD risk factors. The interaction between traditional and non-traditional CVD risk factors results in high morbidity and mortality in SLE patients due to cardiovascular alterations. SLE: systemic lupus erythematosus; TC: total cholesterol; TG: triglycerides; LDL-C: low-density lipoprotein cholesterol; HDL-C: high-density lipoprotein cholesterol; BMI: body mass index; TNF-α: tumor necrosis factor alpha; IL-17: interleukin 17; IFN-1: Type 1 interferons; IL-6: interleukin 6; CRP: C-reactive protein; **ox-LDL:** oxidized low-density lipoprotein; anti-β-2GP1: anti-beta-2 glycoprotein 1 antibodies.

SLE patients present a high prevalence of cardiovascular risk factors; CVD has even been described as one of the main comorbidities and causes of death in SLE. Several studies have shown that SLE patients have a higher prevalence of traditional risk factors, such as obesity, hypertension, and dyslipidemia, compared with the general population; also, the presence of inflammatory molecules related to SLE, such as C-reactive protein (CRP), inflammatory cytokines, and SLE pharmacotherapy, could contribute to CVD development in SLE [20,21]. In particular, genetic susceptibility to SLE has been reported in Mendelian randomization analyses (MRI) to be associated with increased risk of heart failure, ischemic stroke, and venous thromboembolism [22]. Therefore, a complex interaction between traditional and non-traditional CVD risk factors in SLE, resulting in an exacerbated immune response and persistent inflammation, could play a crucial role in CVD in SLE patients [21,23,24], as we shall see later in the following subtopics (Figure 2).

### 3.1. Traditional Risk Factors in SLE Patients

#### 3.1.1. Obesity

Obesity is a chronic inflammatory disease widely linked to CVD in the general population and SLE patients [25]. The connection between obesity and immunity has been widely described; under conditions of excess weight, adipose tissue dysfunction promotes an increase in pro-inflammatory cytokines, such as TNF-α and IL-6 [19].

SLE patients present a high prevalence of excess weight (overweight and obesity), which has been associated with dyslipidemia, atherosclerosis, and higher clinical disease activity than in normal-weight SLE patients [5,25,26].

#### 3.1.2. Dyslipidemia

Dyslipidemia is a common SLE comorbidity; the prevalence of dyslipidemia in these patients can even increase by 60% in the 3 years following diagnosis [27]. SLE patients present higher levels of very low-density lipoprotein-cholesterol (VLDL-C) and TG, and lower HDL-C levels, compared with healthy controls, a phenomenon known as “lupus pattern of dyslipidemia”. Similarly, active SLE patients have higher VLDL-C and TG levels, and lower HDL-C and LDL-C levels than inactive SLE patients (“active lupus pattern of dyslipidemia”) [9]. The exact mechanisms in the relationship between clinical disease activity and dyslipidemia are not fully understood; however, one of the mechanisms suggested is related to an increase in the number of T follicular helper cells, which stimulates the formation of germinal centers and, subsequently, autoantibody production [28].

#### 3.1.3. Insulin Resistance

There is an association between alterations in glucose levels and CVD. IR could lead to micro- or macroangiopathy, peripheral arterial dysfunction, hampered blood flow, hypertension, and cell dysfunction in cardiomyocytes and endothelial cells; as a consequence, IR may increase the risk for coronary artery disease blockage, stroke, and heart failure [29]. Regarding SLE patients in the USA population, it has been reported that the prevalence of IR is higher in this population compared with healthy controls (29.4% and 19.8%, respectively). SLE treatment (glucocorticoids), obesity, and levels of inflammatory markers may explain the disturbances in glucose levels in SLE [30].

#### 3.1.4. Smoking

Reactive oxygen species in cigarette smoke cause oxidative stress and upregulation of inflammatory cytokines, which play a relevant role in endothelial dysfunction by reducing nitric oxide bioavailability; specifically, superoxide anions can reduce nitric oxide availability through the formation of peroxynitrite and are also associated with LDL-C oxidation, which increases the risk of coronary artery disease and stroke. In SLE patients, smoking has been widely associated with CVD and markers of subclinical atherosclerosis; smoking has even been identified as a risk factor for the progression of coronary artery calcification [31].

#### 3.1.5. Hypertension

Hypertension is one of the main risk factors for CVD in the general population, and the SLE population presents with a high prevalence of hypertension [32,33]. The exact mechanism that predisposes SLE patients to hypertension is still unclear; however, some of the mechanisms suggested are the presence of lupus nephritis, the alteration of vascular endothelial function with subsequent endothelial cell activation, and the increase in endothelin 1, causing renal vasoconstriction and water/sodium retention [33,34].

#### 3.1.6. Sedentary Lifestyle

Physical activity reduces CVD risk and other comorbidities, such as obesity and metabolic syndrome (MetS) [35,36]. Conversely, a sedentary lifestyle can promote the morbidity and mortality associated with chronic degenerative diseases [37].

According to a systematic review conducted in populations in the USA, Brazil, UK, Serbia, Sweden, and Belgium, exercise is not contraindicated in SLE, and it has no deleterious effect on clinical disease activity [38]. However, SLE patients have a high prevalence of inadequate physical activity, which could increase the risk of comorbidities in this population [39].

#### 3.1.7. MetS

MetS is considered an independent risk factor for CVD, and women with MetS have a twofold increased risk of experiencing cardiovascular events. In SLE patients, high MetS prevalence could be related to the inflammatory processes attributed to the pathology of SLE, which contributes to the development of alterations linked to MetS [21]. Further, it has been described that MetS predisposes SLE patients to new cardiovascular events and vascular mortality, as well as to the development of chronic kidney disease and diabetes mellitus [40].

### 3.2. SLE Non-Traditional Risk Factors

#### 3.2.1. Hyperhomocysteinemia

Hcy is a nonessential amino acid resulting from methionine transmethylation; a high serum level of this molecule (>15 mmol/L) is considered to be a CVD risk factor, known as hyperhomocysteinemia, and is associated with endothelial damage and vascular disease. One mechanism between CVD and hyperhomocysteinemia is mediated by asymmetric dimethylarginine (ADMA), an endogenous inhibitor of endothelial nitric oxide synthase (eNOS). ADMA causes impaired nitric oxide bioavailability (NO), promoting the uncoupling of eNOS and increasing reactive oxygen species production [41]. SLE patients have elevated levels of Hcy [42,43], which could be related to genetic factors such as methylene tetrahydrofolate reductase (*MTHFR*) genetic variants such as +677 C > T (rs1801133) and +1298 A > C (rs1801131), or to nutritional factors such as poor consumption of folate and cobalamin, which are necessary for Hcy metabolism [5,26,42,44].

#### 3.2.2. Antiphospholipid Antibodies and Complex Immune Damage

Several autoantibodies and immune complexes have been linked to vascular damage and atherosclerosis in SLE; as such, antiphospholipid (aPL) antibodies—including lupus anticoagulant, anticardiolipin, and anti-β2-glycoprotein I antibodies (anti-β-2GP1)—are associated with thrombotic events driven by immune-mediated mechanisms [45,46,47].

The mechanism that links the presence of aPL antibodies to endothelial injury in SLE is not completely clear; possible pathways include anti-β2-GPI binding to β2-GPI and subsequent endothelial cell activation, and the enhanced formation of foam cells induced by ox-LDL/β2-GPI/anti-β2-GPI immune complex formation [45,48].

#### 3.2.3. Pro-Inflammatory Cytokines

Pro-inflammatory cytokines, such as type 1 interferons (IFN-1) and TNF-α, play a relevant role in the development of atherosclerosis; these cytokines can increase levels of chemotactic proteins and adhesion molecules, which promote the recruitment of monocytes and T cells into the endothelial wall [23].

SLE patients present high levels of type 1 IFNs, mainly IFN-α; this cytokine can reduce endothelial progenitor cells, suppress neo-vascularization in sites of endothelial injury, and may contribute to the development of atherogenesis by enhancing foam cell formation, platelet activation, and plaque rupture. TNF-α, another cytokine involved in SLE pathogenesis, can stimulate monocyte differentiation into macrophages and promote foam cell formation; moreover, high TNF-a levels have been associated with high TG and low HDL-C levels [13,49].

#### 3.2.4. C-Reactive Protein

CRP is an acute-phase reactant produced by the liver under the stimulation of IL-6 and IL-1β. It has been proposed that an alteration in CRP production occurs in SLE, which could induce high CRP levels. The altered levels of CRP in SLE could be related its physiopathology or to the presence of some genetic variants, such as −717 A > G (rs2794521), −409 G > A (rs3093062), +1444 C > T (rs1130864), and +1846 C > T (rs1205) in the *CRP* gene [50].

Elevated CRP serum levels are a risk factor for atherosclerosis [51], MetS [52], and obesity [53]. In addition, CRP may modulate CVD by promoting LDL-C phagocytosis by macrophages, inducing apoptosis of endothelial cells, inhibiting angiogenesis, and increasing nuclear factor kappa β (NF-κβ) activation [54].

#### 3.2.5. Lupus Nephritis

Lupus nephritis is a common alteration in SLE and it is mainly responsible for the deposition of immune complexes in the glomeruli and inflammatory tubulointerstitial changes. In general, it has been reported that 16–45% of SLE patients present with lupus nephritis and about 20% will go on to develop end-stage renal disease [55,56]. However, ethnicity could influence renal manifestations; in this sense, there is evidence to suggest that Black, Hispanic, and Asian/Pacific Island SLE patients have an increased prevalence of renal alterations [57]. In addition, lupus nephritis has been described as a CVD risk factor associated with atherosclerosis in SLE [57] and could increase 2.8-fold the risk of myocardial infarction in this population [58].

#### 3.2.6. SLE Pharmacotherapy Associated with Increased CVD Risk

##### Glucocorticoids

Glucocorticoids can alter mechanisms involved in lipid metabolism. Increased lipolysis; increased LPL and adipokine activity; IR; and free fatty acids β-oxidation inhibition are some of the mechanisms associated with glucocorticoid use [59]. Chronic prednisone administration may influence the development of atherogenic dyslipidemia, mediated by an increase in hepatic lipid synthesis and its mobilization in peripheral tissues. It has been shown that prednisone dose is positively correlated with total serum cholesterol, LDL-C, TG, glucose, arterial blood pressure, and BMI [8]. Moreover, a prednisone dose ≥ 30 mg/day has been correlated with increased serum total cholesterol and TG [9,59].

##### Methotrexate

Methotrexate is a cytotoxic agent and a folic acid antagonist that depletes intracellular folate levels by the inhibition of key enzymes involved in folate and de novo nucleotide synthesis pathways, such as the Dihydrofolate Reductase (DHFR) and Thymidylate Synthase (TYMS) [60,61]. This inhibition subsequently affects several metabolic pathways, such as the Hcy–methionine pathway [60]. Therefore, methotrexate administration at doses of 10–25 mg per week have been associated with elevated levels of Hcy in rheumatoid arthritis and SLE patients [49,60], which may be counteracted by folic acid supplementation [60].

#### 3.2.7. SLE Pharmacotherapy Associated with Decreased CVD Risk

##### Antimalarials

Antimalarial drugs such as hydroxychloroquine (HCQ) and chloroquine (CQ) have long been used to treat inflammatory rheumatism and SLE; however, their mechanisms of action remain unclear. The mechanism suggested is that these molecules, which are weak bases, interfere with the phagocytosis through an increase in pH in intracellular compartments, disrupting the selective presentation of self-antigens and blocking the proliferative responses of T-cells [62].

Antimalarials, mainly HCQ, have been associated with decreased dyslipidemia [45,62,63], diabetes, and thrombotic events in SLE patients [62]. The mechanism proposed is that antimalarials promote upregulation of the LDL-C receptors, with subsequent enhancement of plasma removal of this lipoprotein and a reduction in hepatic cholesterol synthesis. In animal models, the decrease in LDL-C was explained by the inhibition of lysosomal function, which led to an accumulation of LDL in the lysosome [62,64,65].

##### Mycophenolate Mofetil

Mycophenolate mofetil (MMF) is another drug used in SLE patients. It is related to low Hcy levels through its ability to decrease the export of Hcy by human proximal tubule epithelial cells, according to an in vitro study [66]. Another mechanism suggested for the way in which MMF influences Hcy metabolism is that mycophenolic acid, an active metabolite of MMF, inhibits the enzyme inosine 5′-monophosphate dehydrogenase; this enzyme participates in purine nucleotide biosynthesis, which is necessary for cell proliferation. The inhibition of cell proliferation causes a decrease in protein turnover and can decrease Hcy levels [66].

## 4. Potential Therapeutic Effect of Nutrients in SLE

Environmental factors, such as diet, have a key role in the progress of autoimmune diseases. Diet has become relevant, not only because of its impact on general health but also because nutrients have immunomodulatory, anti-inflammatory, and antioxidant properties that can determine the evolution of several diseases. In this context, diet and nutritional status are modifiable environmental factors involved in SLE pathophysiology that could modulate the response to treatment and the clinical outcomes of the disease [12,15,18]. Therefore, non-pharmacological therapy such as diet might be a safe additional strategy for SLE treatment.

Excess weight and dyslipidemia in SLE patients have been described as associated with clinical disease activity, organ damage, and increased CVD risk [15,17]. In contrast, the potential beneficial role of some nutrients, such as polyunsaturated fatty acids, vitamins A, C, D, and B, and selenium on SLE pathogenesis has been reported in murine models and humans [5,67] (Table 1). Additionally, it has been consistently described that the intake of some nutrients, such as dietary fiber and PUFAs, are directly related to the modulation of traditional risk factors, such as dyslipidemia and hypertension in SLE [68,69]. Likewise, the intake of vitamins B9 and B12 regulate some non-traditional risk factors, such as hyperhomocysteinemia [70,71].

### 4.1. Energy (Calories)

Calorie restriction of around 30–40% may delay expression and ameliorate the progression of autoimmune diseases in the murine model (NZB/NZW) [16]. In the same way, energy restriction maintains CD8+ T and decreases CD4+ T lymphocytes; reduces IL-12, IFN-γ, IgA, and IgG production; delays the onset of kidney disease; and increases life expectancy in response to the downregulation of NF-κβ mRNA expression [16]. Otherwise, restricted consumption of specific amino acids, such as phenylalanine and tyrosine, and a moderate protein intake improves renal function in SLE patients [78]. This improvement is related to the fact that the accumulation of kynurenine (a tryptophan metabolite) and the depletion of cysteine and glutathione in SLE promote the activation of the mechanistic target to rapamycin (mTOR) inflammatory pathway [79]. Therefore, a possible way to reduce inflammation in SLE patients could be the use of mTOR inhibitors, such as acetylcysteine or rapamycin [80,81]. However, because of their side effects they are not the first option for intervention; therefore, calorie restriction could be a safer strategy for reduction in mTOR hyperactivity.

### 4.2. Polyunsaturated Fatty Acids, PUFA (n-3 and n-6)

Dietary lipids, especially polyunsaturated fatty acids, provide energy and are constituents of different tissues. A balance in the consumption of EPA and DHA (3:1 ratio) together with energy restriction has demonstrated a greater anti-inflammatory capacity, reducing the atherogenic lipid profile and reducing the severity of autoimmunity and nephritis compared with a calorie-restricted diet alone, or a PUFA-rich diet alone [82,83,84].

Principal food sources of the PUFAs *n*-6, and *n*-3 are oils from plants and seeds—such as corn, soybean, flaxseed—and fish oil [85]. Supplementation with fish oil has been associated with macrophage activity suppression and reduction in pro-inflammatory cytokine expression and cyclooxygenase metabolites, which contribute to the reduction in clinical disease activity and proteinuria and preserve adequate glomerular filtration in animal and human studies [12,86,87]. Moreover, it has been reported that flaxseed inhibits platelet-activating factor, which tends to be elevated in SLE; a daily dosage of 30 g/day appears to reduce total cholesterol by 11% and LDL-C serum levels by 12% [78].

On the other hand, DHA inhibits the action of NF-κβ and TNF-α; reduces anti-double stranded DNA antibody (anti-dsDNA) titers; ameliorates the deposition of IgG in the kidneys; and reduces IL-18 in the NZB/NZW mouse model [88,89]. At the same time, EPA has been demonstrated to reduce IL-1β, IL-6, and TNF-α production, changing cell membrane composition and inhibiting the interaction between receptors and inflammatory cytokines [15,82]. Therefore, PUFAs are considered pro-resolving lipid mediators and have been associated with an enhancement in plaque stability and a reduction in atherosclerosis [90,91].

### 4.3. Vitamin A

Vitamin A (retinoic acid) is an essential micronutrient for the optimal performance of the immune system; it is the active form of all-trans-retinoic acid (ATRA), a nuclear transcription factor that regulates gene transcription and expression [18,92]. Retinoic acid increases the proliferation and cytotoxicity of T cells, specifically regulatory T cells, and inhibits Th17 cell proliferation in murine models [73,92]. The increased ingestion of retinoids has been associated with improving lupus nephritis and proteinuria and with reducing levels of anti-dsDNA in humans and murine studies [73,93]. In female humans, a dose of 100,000 IU of vitamin A daily per two weeks was associated with an immune response improvement [94]; nevertheless, the excessive consumption of vitamin A (>100,000 IU) has been related to anemia, headache, dry skin, nausea, and even death [15,78].

### 4.4. Antioxidant Vitamins (C and E)

Vitamins such as C and E are beneficial in treating SLE because of their antioxidant properties. Vitamin C acts as an immunomodulator, releasing anti-inflammatory mediators [95]. In SLE patients, vitamin C consumption has been inversely associated with clinical disease activity, as vitamin C reduces oxidative stress and suppresses auto-antibody production [70]. On the other hand, a study conducted on SLE women showed that vitamin E supplementation suppressed anti-dsDNA antibodies production. However, no differences were found in DNA oxidative damage, which suggested that vitamin E may decrease autoantibody production through a mechanism independent of its antioxidant activity [96].

### 4.5. B Vitamins

B vitamins can act as coenzymes in two reactions: the remethylation of Hcy to methionine and the isomerization of l-methylmalonyl-CoA; the first reaction occurs in the cytosol and is catalyzed by the methionine synthase enzyme, followed by a l-methylmalonyl-CoA mutase catalysis in the mitochondria [71]. B vitamin deficiency is linked with many pathological conditions, such as anemia, megaloblastosis, neuropathy, and neurologic disorders, theoretically linked with hyperhomocysteinemia [15,71].

High levels of homocysteine increase the risk of CVD in patients with SLE; thus, high consumption of vitamins B6, B12, and folate (vitamin B9) may be recommended to promote homocysteine remethylation [70,71]. Supplementation with folate has been associated with SLE symptom improvement and longer life expectancy in a study conducted in mice [97]. Furthermore, a study conducted in SLE children with dyslipidemia showed that niacin (vitamin B3) consumption reduced TG and LDL-C levels [70,98].

### 4.6. Vitamin D

Vitamin D has been implicated in pleiotropic biological functions such as mineral (calcium phosphates) homeostasis and immunomodulation [99,100]. Its deficiency (<20 ng/mL) has been associated with many diseases, including infectious and autoimmune diseases [101].

The immunomodulatory effect of vitamin D has been reported through the activity by various mechanisms of its active metabolite calcitriol (1,25(OH)D_3_). In in vitro dendritic cells (DCs), the presence of calcitriol keeps DCs in a tolerogenic state; these tolerogenic DCs (tDCs) decrease levels of pro-inflammatory mediators, such as IL-12 and TNF-α, and increase IL-10 production, promoting polarization of T regulatory (Treg) cells [102,103].

A negative correlation has been described between vitamin D (calcidiol) serum levels and clinical disease activity in SLE patients [5,100]. Moreover, vitamin D supplementation in SLE patients (2000, 4000, or 5000 IU weekly for 6 months) caused an increase in Foxp3 expression and a decrease in Th17 cells, thus improving endothelial function [104,105,106]. However, the evidence is still inconclusive; some studies do not support the positive effect of vitamin D supplementation on CVD risk; observational studies have shown that not only calcidiol deficiency, but also very high calcidiol serum levels, could be a risk factor for the development of CVD [107].

### 4.7. Selenium

Selenium is an essential trace element and micronutrient required for numerous aspects of human health. Several of its biological effects occur through the incorporation of selenoproteins, many of which are involved in the activation, proliferation, and differentiation of adaptative and innate immune cells. A study conducted on SLE mouse models showed that selenium supplementation prolongs the life of these mice and promotes the inhibition of activation, differentiation, and maturation of B cells and macrophages, which subsequently reduced anti-dsDNA antibodies. In this regard, such activity could suggest a potential therapeutic selenium supplementation for SLE patients [77].

## 5. Nutrients to Target Cardiovascular Disease Risk in SLE Patients

**Figure 3:**
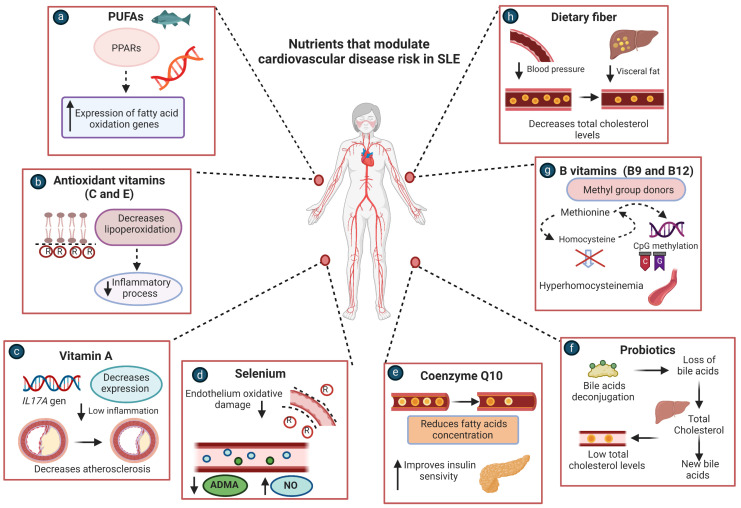
Nutrients that modulate the cardiovascular disease risk in SLE patients. (**a**) PUFAs: can reduce triglycerides through activation of PPARs to increase expression of genes involved in fatty acid oxidation. (**b**) Antioxidants vitamins C and E: can decrease lipoperoxidation and reduce superoxide and hydrogen peroxide generation linked to CVD. (**c**) Vitamin A: decreases expression of the *IL17A* gene, which could reduce the inflammatory process and slow atherosclerosis progression. (**d**) Selenium: reduces ADMA concentration, which is considered an independent cardiovascular risk factor due to its capacity to inhibit NO production. (**e**) Coenzyme Q10: can improve the lipid profile and reduce insulin resistance through modulation of insulin and adiponectin receptors. (**f**) Probiotics: can deconjugate bile acids which coprecipitate with total cholesterol, to compensate for the loss of bile acids; the liver then converts cholesterol into new bile acids, which can reduce serum total cholesterol levels. (**g**) B vitamins (B12 and B9): act as cofactors in Hcy metabolism and promote its conversion to methionine, thus decreasing Hcy serum levels. (**h**) Dietary fiber: its fermentation by gut bacteria provides short-chain fatty acids, such as propionic acid; its absorption decreases cholesterol synthesis in the liver and increases water and sodium absorption into the colonic mucosal cells. SLE: systemic lupus erythematosus; PUFAs: polyunsaturated fatty acids; PPARs: peroxisome proliferator-activated receptors; CVD: cardiovascular disease; ADMA: asymmetric dimethylarginine NO: nitric oxide; Hcy: homocysteine.

Clinical trials and observational studies have provided evidence about the effects of some nutrient and micronutrient consumption or supplementation on CVD risk factors in SLE [108], as well as in other chronic diseases [109]; it could, therefore, provide a non-pharmacological therapy for SLE patients (Figure 3).

### 5.1. PUFAs

PUFAs are nutrients widely associated with decreasing dyslipidemia in the general population; evidence shows that PUFAs can reduce triglycerides by activating peroxisome proliferator-activated receptors (PPARs) and increase the expression of genes involved in fatty acid oxidation [68]. Randomized interventional trials on SLE patients have reported a decrease in clinical disease activity and an improvement in endothelial function [110,111] (Table 2). The anti-inflammatory effect of omega-3 fatty acid supplementation has been demonstrated in several clinical trials; it has been shown to significantly reduce inflammatory marker levels and improve both lipid profile and endothelial function [108]. This evidence suggests PUFAs (mainly omega-3 supplementation) as a possible strategy to target inflammation and CVD in SLE. In particular, it has been described that serum concentrations of PUFA precursors, such as linoleic acid (LA) and α-linolenic acid, are significantly higher in SLE patients compared with healthy controls. Likewise, the concentration of LA has been positively correlated with antinuclear antibody (ANA) titers and doses of corticosteroids; eicosapentaenoic acid (EPA) and docosahexaenoic acid (DHA) were inversely correlated with anti-dsDNA antibody concentration. Both *n*-6 and *n*-3 PUFA precursors may participate in the inflammatory process in SLE patients [112].

### 5.2. Antioxidant Vitamins (C and E)

In SLE, oxidative stress is attributed to the inflammatory process characteristic of the disease, which can induce lipoperoxidation and contributes to the superoxide and hydrogen peroxide production linked to CVD [120,121]. The evidence suggests that vitamin C and E supplementation can reduce lipoperoxidation in SLE patients; however, low consumption and serum levels of these vitamins have been reported in this population [113].

### 5.3. B Vitamins

There is evidence that B vitamins could influence the lipid profile; B6 supplementation in hypertriglyceridemic male patients can reduce total plasma cholesterol, which is attributed to the necessity of B6 for endogenous carnitine synthesis; it has even been reported that a vitamin B6-deficient diet reduces carnitine biosynthesis [122]. The insufficient consumption of B vitamins is also related to increased homocysteine levels, which can induce the development of CVD [26]. In this sense, some studies have shown that complex B supplementation reduces homocysteine levels and decreases the thickness of the carotid artery intima-media wall; therefore, complex B supplementation could be a therapeutic alternative to hyperhomocysteinemia in SLE patients [109,114].

### 5.4. Coenzyme Q10

The coenzyme Q10 (coQ10) is a lipophilic benzoquinone that plays a relevant role in the mitochondrial electron transport chain. It has been reported that coQ10 has beneficial metabolic effects, and the evidence shows that it can improve the lipids profile and reduce insulin resistance. Because of this, coQ10 supplementation has been suggested as a therapeutic alternative for dyslipidemia [115].

### 5.5. Probiotics

Studies in humans have shown a possible link between probiotics and improvement in the lipid profile. The mechanism suggested is that some bacteria, such as *L. acidophilus*, deconjugate bile acids, which coprecipitate with cholesterol at a pH < 5.5, to compensate for the loss of bile acids; the liver then converts cholesterol into new bile acids, thus reducing cholesterol levels [116].

### 5.6. Dietary Fiber

Dietary fiber is a non-digestible form of carbohydrates; it can be classified either as soluble or insoluble fiber, according to its water solubility properties. Water-insoluble fibers mainly include lignin, cellulose, and hemicellulose, while water-soluble fibers include pectin, gums, and mucilage. The soluble fiber provides a source of short-chain fatty acids, such as propionic acid, which are absorbed from the large intestine; their absorption decreases cholesterol synthesis in the liver and increases sodium and water absorption into the colonic mucosal cells; this could be the mechanism through which dietary fiber has been associated with a reduction in some CVD risk factors, such as systolic blood pressure, visceral adiposity, total cholesterol, and LDL-C serum levels [69,117].

### 5.7. Vitamin A

Vitamin A plays a crucial role in immune responses; in vitro studies have shown a strong suppressive effect on pro-inflammatory Th17 cell function [92]. A clinical trial conducted on atherosclerotic patients who were not taking any medication or suffering from immune disease was randomly assigned to receive either vitamin A or placebo. The results showed that vitamin A supplementation decreased the expression of the IL-17 gene [118]; therefore, it could reduce the inflammatory process and the progression of atherosclerosis in SLE patients.

### 5.8. Selenium

Selenium is an essential micronutrient with antioxidant properties. It has also been suggested that selenium can reduce CVD risk by preventing endothelium oxidative damage. Evidence shows that selenium supplementation reduces ADMA concentration, which is considered an independent cardiovascular risk factor owing to its capacity to inhibit NO production [119].

## 6. Limitations and Perspectives

The present study is a literature review; therefore, the search strategy was different from a systematic review. Furthermore, the article selection strategies are not reproducible. On the other hand, some functional foods have demonstrated positive effects on alterations related to CVD and SLE pathogenesis, and they might not have been considered in this study, which was focused on anti-inflammatory and antioxidant properties of micronutrients. Nevertheless, this review provides a qualitative synthesis of the historical and recent evidence about nutrient and micronutrient effects on CVD risk factors and their possible use to target cardiovascular alterations in SLE. Additionally, more cohort studies are necessary to evaluate the risk/benefit ratio of long-term vitamin supplementation in SLE and determine the doses necessary to impact cardiovascular health. It is well known that mega-doses of fat-soluble vitamins, such as A, D, E, and K, may cause toxicity. Therefore, there should be consideration of the upper dose limit in their administration and the presence of serum deficiency, to determine the pharmacological or dietary approaches for their correction and to establish dietary strategies for cardiovascular disease treatment.

## 7. Conclusions

CVD is the main complication and cause of death in SLE. The high prevalence of traditional and non-traditional cardiovascular risk factors places this population at high risk of morbidity and mortality for CVD. Based on this, it is vital to identify early CVD risk factors in the SLE population and find alternatives to prevent or reverse them. Studies in animal models and humans have shown that some nutrients positively impact SLE pathogenesis. Moreover, nutrients with immunomodulatory and antioxidant properties could decrease the inflammatory process and indirectly prevent the development of CVD and pro-inflammatory events. Notably, some clinical trials conducted in SLE patients have shown the positive effect of nutritional supplementation on CVD alterations. Therefore, nutrients could provide a successful and safe alternative to reduce CVD risk in SLE; however, more clinical trials are necessary to suggest a cardioprotective diet for SLE patients.

## Figures and Tables

**Table 1:** Nutrients with potential beneficial effects in SLE pathophysiology: evidence in animal and human studies.

Dietary Component	Model System	Pathophysiological Process	Effects	Reference
Polyunsaturated fatty acids (EPA and DHA)	Weanling female (NZB × NZW) F_1_(B/W) mice	Reduction in IL-1*β*, TNF-*α*, and ICAM-1 expression	Delay the onset and progression of lupus nephritis	[72]
Vitamin A	NZB/WF mice	Reduction in IFN-γ, IL-2, and anti-DNA levels. Reduction in glomerular deposits of IgG2a	Alleviates autoimmune tissue injuries and prolongs survival	[73]
Vitamin C, E and *β* carotene	MRL/lpr mice	Decrease IgG and anti-dsDNA levels	Possible decrease in SLE symptoms	[74]
B vitamins	C57BL/6 mice	Decrease CD40L expression and hematuria	Ameliorate SLE disease	[75]
Vitamin D	Premenopausal women with SLE	Increases the number of T-reg cells and reduces the number of CD8+ CD28- T cells	Lowers clinical disease activity	[76]
Selenium	NZB/NZW-F1 mice	Inhibits activation, differentiation, and maturation of B cells and macrophages, reduction in autoantibodies to dsDNA	Lowers clinical disease activity	[77]

SLE: systemic lupus erythematosus, EPA: eicosapentaenoic acid; DHA: docosahexaenoic acid; IL-interleukin; TNF-α: tumor necrosis factor alpha, (NZB × NZW) F1(B/W): New Zealand Mixed Mice; MRL/lpr mice: MLR lymphoproliferation strain; C57BL/6 mice; anti-dsDNA: anti-double stranded DNA antibodies.

**Table 2:** Nutrients and their potential therapeutic effects on CVD risk in SLE patients and other patients with cardiometabolic pathologies: evidence from clinical studies.

Source	Study Group	Effective Dose and Duration	The Expected Outcome in CVD	Reference
***n*-3 PUFA**	Randomized interventional trial in 69 SLE patients from Northern Ireland.	3 mg for 24 weeks Omacor (omega-3 acid ethyl esters) 4 capsules per day provided 1.8 g EPA and 1.2 g DHA.	Decrease in clinical disease activity by reduction in SLAM-R score from 9.4 to 6.3. Improved endothelial function by increasing flow-mediated dilatation from 3 to 5.7%.	[111]
	A comparative observational study of 62 SLE patients from Brazil.	Fish oil 3 g/day for 120 days.	Increased adiponectin levels and decreased leptin levels.	[110]
**Vitamin C and E**	A double-blind placebo-controlled pilot study in 39 SLE patients from Hong Kong.	Pill with 500 mg of vitamin C and 800 UI vitamin E (D-α tocopherol succinate) for 12 weeks.	Decreased lipid peroxidation measured by a reduction in malondialdehyde concentration.	[113]
**B vitamins**	A randomized, double-blind, placebo-controlled trial in 8171 women with coronary risk factors from the USA	Combination pill containing 2.5 mg of folic acid, 50 mg of B6, and 1 mg of B12	Decrease in geometric mean homocysteine levels by 18.5%.	[109]
	A randomized, double-blind, placebo-controlled trial in 186 patients with end-stage kidney disease from Brazil.	Oral folic acid 10 mg, 3 times a week for 2 years.	Decrease in carotid artery intima-media wall thickness from 1.94 ± 0.59 mm to 1.67 ± 0.38 mm. Decrease in homocysteine levels from 25 µmol/L to 10.5 µmol/L.	[114]
**Coenzyme Q10**	A randomized, double-blind, placebo-controlled trial in 101 subjects with dyslipidemia.	30 mg/day of coQ10 for 24 weeks.	Decrease in LDL-C by 6.5%, triglycerides by 19.90%, and serum insulin by 21.09%.	[115]
**Probiotics**	Cross-over study in 29 women with and without hypercholesterolemia from Germany.	300 g/day of yogurt for 21 weeks.	Increase in HDL-C concentration by 0.3 mmol/L. The ratio of LDL-C/HDL-C decreased from 3.24 to 2.48.	[116]
**Dietary fiber**	A randomized, double-blind, placebo-controlled trial in 91 type 2 diabetic patients.	30 g/day of soluble fiber (Gum Arabic) for 3 months.	Decrease in systolic blood pressure by 7.6% and decrease in visceral adiposity index by 23.7%.	[69]
	A randomized, double-blind, placebo-controlled trial in 80 mildly hypercholesterolemic Asian Indians.	3 g of soluble fiber from 70 g/day of oats.	Decrease in cholesterol levels by 8.1% and decrease in LDL-C levels by 11.6%.	[117]
**Vitamin A**	A double-blind study in 31 atherosclerotic patients and 15 healthy controls.	25,000 IU/day of retinyl palmitate for 4 months.	Decrease in IL-17 gene expression by 0.63-fold in fresh cells and 0.82-fold in PHA-activated T cells.	[118]
**Selenium**	A randomized, double-blind, placebo-controlled trial in 66 women with polycystic ovary syndrome.	200 µg/day of selenium for 12 weeks.	ADMA concentration decreased from 85.14 ± 75 to 56.4 ± 38.6 ng/L.	[119]

CVD: cardiovascular disease, SLE: systemic lupus erythematosus, PUFA: polyunsaturated fatty acid; SLAM-R: Systemic Lupus Activity Measure-Revised; EPA: eicosapentaenoic acid; DHA: docosahexaenoic acid; LDL-C: low-density lipoprotein cholesterol; HDL-C: high-density lipoprotein cholesterol. ADMA: asymmetric dimethylarginine.

## Data Availability

Not applicable.

## Abbreviations

SLE	Systemic lupus erythematosus
CVD	cardiovascular disease
CRP	C-reactive protein
Hcy	homocysteine
IR	insulin resistance
Anti-oxLDL	antibodies against oxidized LDL
MetS	Metabolic syndrome
SLEDAI	Systemic Lupus Erythematosus Disease Activity Index
ADMA	Asymmetric dimethylarginine
MTHFR	Methylene tetrahydrofolate reductase
aPL	Antiphospholipid
IFNs	interferons
NF-κβ	Nuclear factor kappa beta
HCQ	Hydroxychloroquine
CQ	Chloroquine
MMF	Mycophenolate mofetil
PUFA	Polyunsaturated fatty acid
CoQ10	Coenzyme Q10
NO	nitric oxide

## References

[1] Ye Y., Wu T., Zhang T., Han J., Habazi D., Saxena R., Mohan C. (2019). Elevated oxidized lipids, anti-lipid autoantibodies and oxidized lipid immune complexes in active SLE. Clin. Immunol..

[2] Sinicato N.A. (2013). Risk Factors in Cardiovascular Disease in Systemic Lupus Erythematosus. Curr. Cardiol. Rev..

[3] Wigren M., Nilsson J., Kaplan M.J. (2015). Pathogenic immunity in systemic lupus erythematosus and atherosclerosis: Common mechanisms and possible targets for intervention. J. Intern. Med..

[4] Croca S., Rahman A. (2017). Atherosclerosis in systemic lupus erythematosus. Best Pract Res. Clin. Rheumatol..

[5] Meza-Meza M.R., Vizmanos-Lamotte B., Muñoz-Valle J.F., Parra-Rojas I., Garaulet M., Campos-López B., Montoya-Buelna M., Cerpa-Cruz S., Martínez-López E., Oregon-Romero E. (2019). Relationship of Excess Weight with Clinical Activity and Dietary Intake Deficiencies in Systemic Lupus Erythematosus Patients. Nutrients.

[6] Hak A.E., Karlson E.W., Feskanich D., Stampfer M.J., Costenbader K.H. (2009). Systemic lupus erythematosus and the risk of cardiovascular disease: Results from the nurses’ health study. Arthritis Rheum..

[7] Manzi S., Meilahn E.N., Rairie J.E., Conte C.G., Medsger T.A., Jansen-McWilliams L., D’Agostino R.B., Kuller L.H. (1997). Age-specific Incidence Rates of Myocardial Infarction and Angina in Women with Systemic Lupus Erythematosus: Comparison with the Framingham Study. Am. J. Epidemiol..

[8] Karp I., Abrahamowicz M., Fortin P.R., Pilote L., Neville C., Pineau C.A. (2008). Esdaile JM. Recent corticosteroid use and recent disease activity: Independent determinants of coronary heart disease risk factors in systemic lupus erythematosus?. Arthritis Rheum..

[9] Szabó M.Z., Szodoray P., Kiss E. (2017). Dyslipidemia in systemic lupus erythematosus. Immunol. Res..

[10] Campos-López B., Meza-Meza M.R., Parra-Rojas I., Ruiz-Ballesteros A.I., Vizmanos-Lamotte B., Muñoz-Valle J.F., Montoya-Buelna M., Cerpa-Cruz S., Bernal-Hernández L.E., De la Cruz-Mosso U. (2021). Association of cardiometabolic risk status with clinical activity and damage in systemic lupus erythematosus patients: A cross-sectional study. Clin. Immunol..

[11] de Miranda Moura dos Santos F., Borges M.C., Telles R.W., Correia M.I.T.D., Lanna C.C.D. (2013). Excess weight and associated risk factors in patients with systemic lupus erythematosus. Rheumatol. Int..

[12] La Cava A. (2019). The Influence of Diet and Obesity on Gene Expression in SLE. Genes.

[13] Svenungsson E., Gunnarsson I., Fei G.Z., Lundberg I.E., Klareskog L., Frostegård J. (2003). Elevated triglycerides and low levels of high-density lipoprotein as markers of disease activity in association with up-regulation of the tumor necrosis factor α/tumor necrosis factor receptor system in systemic lupus erythematosus: Blood Lipids and TNFα Activity in SLE. Arthritis Rheum..

[14] Oeser A., Chung C.P., Asanuma Y., Avalos I., Stein C.M. (2005). Obesity is an independent contributor to functional capacity and inflammation in systemic lupus erythematosus. Arthritis Rheum..

[15] Klack K., Bonfa E., Neto E.F.B. (2012). Diet and nutritional aspects in systemic lupus erythematosus. Rev. Bras. Reumatol..

[16] Muthukumar A., Zaman K., Lawrence R., Barnes J.L., Fernandes G. (2002). Food Restriction and Fish Oil Suppress Atherogenic Risk Factors in Lupus-Prone (NZB × NZW) F1 Mice. J. Clin. Immunol..

[17] Borges M.C., dos Santos F.D.M.M., Telles R.W., Lanna C.C.D., Correia M.I.T. (2012). Nutritional status and food intake in patients with systemic lupus erythematosus. Nutrition.

[18] Aparicio-Soto M., Sánchez-Hidalgo M., Alarcón-de-la-Lastra C. (2017). An update on diet and nutritional factors in systemic lupus erythematosus management. Nutr. Res. Rev..

[19] Versini M., Jeandel P., Rosenthal E., Shoenfeld Y. (2014). Obesity in autoimmune diseases: Not a passive bystander. Autoimmun. Rev..

[20] Sagar D., Gaddipati R., Ongstad E.L., Bhagroo N., An L.L., Wang J., Belkhodja M., Rahman S., Manna Z., Davis M.A. (2020). LOX-1: A potential driver of cardiovascular risk in SLE patients. PLoS ONE.

[21] Zeller C., Appenzeller S. (2008). Cardiovascular Disease in Systemic Lupus Erythematosus: The Role of Traditional and Lupus Related Risk Factors. Cur. Cardiol. Rev..

[22] Gao N., Kong M., Li X., Wei D., Zhu X., Hong Z., Ni M., Wang Y., Dong A. (2022). Systemic Lupus Erythematosus and Cardiovascu-lar Disease: A Mendelian Randomization Study. Front. Immunol..

[23] Kahlenberg J.M., Kaplan M.J. (2011). The interplay of inflammation and cardiovascular disease in systemic lupus erythematosus. Arthritis Res. Ther..

[24] Mercurio V., Lobasso A., Barbieri L., Parrella P., Ciervo D., Liccardo B., Bonaduce D., Tocchetti C.G., De Paulis A., Rossi F.W. (2019). Inflammatory, serological and vascular determinants of cardiovascular disease in systemic lupus erythematosus patients. Int. J. Mol. Sci..

[25] Rizk A., Gheita T.A., Nassef S., Abdallah A. (2012). The impact of obesity in systemic lupus erythematosus on disease parameters, quality of life, functional capacity and the risk of atherosclerosis: Obesity in SLE. Int. J. Rheum. Dis..

[26] Salomão R.G., de Carvalho L.M., Izumi C., Czernisz É.S., Rosa J.C., Antonini S.R.R., Bueno A.C., Almada M.O.R.D.V., Coelho-Landell C.D.A., Jordão A.A. (2018). Homocysteine, folate, hs-C-reactive protein, tumor necrosis factor alpha and inflammatory proteins: Are these biomarkers related to nutritional status and cardiovascular risk in childhood-onset systemic lupus erythematosus?. Pediatr. Rheumatol. Online J..

[27] Urowitz M.B., Gladman D., Ibañez D., Fortin P., Sanchez-Guerrero J., Bae S., Clarke A., Bernatsky S., Gordon C., Hanly J. (2008). Accumulation of coronary artery disease risk factors over three years: Data from an international inception cohort. Arthritis Rheum..

[28] Ryu H., Chung Y. (2018). Dyslipidemia promotes germinal center reactions via IL-27. BMB Rep..

[29] Patel R., Dwivedi M., Mansuri M.S. (2016). Association of Neuropeptide-Y (NPY) and Phenotype Correlation and Plasma Lipids with Type-II Diabetes. PLoS ONE.

[30] Chung C.P., Avalos I., Oeser A., Gebretsadik T., Shintani A., Raggi P., Stein C.M. (2007). High prevalence of the metabolic syndrome in patients with systemic lupus erythematosus: Association with disease characteristics and cardiovascular risk factors. Ann. Rheum. Dis..

[31] Giannelou M., Mavragani C.P. (2017). Cardiovascular disease in systemic lupus erythematosus: A comprehensive update. J. Autoimmun..

[32] Bateman B.T., Shaw K.M., Kuklina E.V., Callaghan W.M., Seely E.W., Hernández-Díaz S. (2012). Hypertension in Women of Reproductive Age in the United States: NHANES 1999-2008. PLoS ONE.

[33] Munguia-Realpozo P., Mendoza-Pinto C., Sierra Benito C., Escarcega R.O., Garcia-Carrasco M., Mendez Martinez S., Etchegaray Morales I., Galvez Romero J.L., Ruiz-Arguelles A., Cervera R. (2019). Systemic lupus erythematosus and hypertension. Autoimmun. Rev..

[34] Ciołkiewicz M., Kuryliszyn-Moskal A., Klimiuk P.A. (2010). Analysis of correlations between selected endothelial cell activation markers, disease activity, and nailfold capillaroscopy microvascular changes in systemic lupus erythematosus patients. Clin. Rheumatol..

[35] Bell J.A., Hamer M., David Batty G., Singh-Manoux A., Sabia S., Kivimaki M. (2014). Combined effect of physical activity and leisure time sitting on long-term risk of incident obesity and metabolic risk factor clustering. Diabetologia.

[36] Reddigan J.I., Ardern C.I., Riddell M.C., Kuk J.L. (2011). Relation of physical activity to cardiovascular disease mortality and the influence of cardiometabolic risk factors. Am. J. Cardiol..

[37] Kohl H.W., Craig C.L., Lambert E.V., Inoue S., Alkandari J.R., Leetongin G., Kahlmeier S., Andersen L.B., Bauman A.E., Blair S.N. (2012). The pandemic of physical inactivity: Global action for public health. Lancet.

[38] O’Dwyer T., Durcan L., Wilson F. (2017). Exercise and physical activity in systemic lupus erythematosus: A systematic review with meta-analyses. Semin. Arthritis Rheum..

[39] Margiotta D.P.E., Basta F., Dolcini G., Batani V., Vullo M.L., Vernuccio A., Navarini L., Afeltra A. (2018). Physical activity and sedentary behavior in patients with Systemic Lupus Erythematosus. PLoS ONE.

[40] Mok C.C. (2019). Metabolic syndrome and systemic lupus erythematosus: The connection. Expert. Rev. Clin. Immunol..

[41] Kumar A., Palfrey H.A., Pathak R., Kadowitz P.J., Gettys T.W., Murthy S.N. (2017). The metabolism and significance of homocysteine in nutrition and health. Nut. Metab..

[42] Giannelou M., Nezos A., Fragkioudaki S., Kasara D., Maselou K., Drakoulis N., Ioakeimidis D., Moutsopoulos H.M., Mavragani C.P. (2018). Contribution of MTHFR gene variants in lupus related subclinical atherosclerosis. Clin. Immunol..

[43] Sam N.B., Zhang Q., Li B.Z., Li X.M., Wang D.G., Pan H.F., Ye D.Q. (2020). Serum/plasma homocysteine levels in patients with systemic lupus erythematosus: A systematic review and meta-analysis. Clin. Rheumatol..

[44] Timlin H., Manno R., Douglas H. (2019). Hyperhomocysteinemia and Lupus Nephritis. Cureus.

[45] Kostopoulou M., Nikolopoulos D., Parodis I., Bertsias G. (2020). Cardiovascular Disease in Systemic Lupus Erythematosus: Recent data on epidemiology, risk factors and prevention. Curr. Vasc. Pharmacol..

[46] Schreiber K., Sciascia S., De Groot P.G., Devreese K., Jacobsen S., Ruiz-Irastroza G., Salmon J.E., Shoenfeld Y., Shovman O., Hunt B.J. (2018). Antiphospholipid syndrome. Nat. Rev. Dis. Primers..

[47] Tektonidou M.G., Laskari K., Panagiotakos D.B., Moutsopoulos H.M. (2009). Risk factors for thrombosis and primary thrombosis prevention in patients with systemic lupus erythematosus with or without antiphospholipid antibodies. Arthritis Rheum..

[48] Zhang X., Xie Y., Zhou H., Xu Y., Liu J., Xie H., Yan J. (2014). Involvement of TLR4 in oxidized LDL/β2GPI/Anti-β2GPI-induced transformation of macrophages to foam cells. J. Atheroscler. Thromb..

[49] Rho Y.H., Chung C.P., Oeser A., Solus J., Raggi P., Gebretsadik T., Shintani A., Stein C.M. (2008). Novel cardiovascular risk factors in premature coronary atherosclerosis associated with systemic lupus erythematosus. J. Rheumatol..

[50] Atisha-Fregoso Y., Lima G., Carrillo-Maravilla E., Posadas-Sánchez R., Pérez-Hernández N., Baños-Peláez M., Iturralde-Chávez A., Hernández-Díaz N., Jakez-Ocampo J., Rodríguez-Pérez J.M. (2018). C-reactive protein (CRP) polymorphisms and haplotypes are associated with SLE susceptibility and activity but not with serum CRP levels in Mexican population. Clin. Rheumatol..

[51] Momiyama Y., Ohmori R., Fayad Z.A., Kihara T., Tanaka N., Kato R., Taniguchi H., Nagata M., Nakamura H., Ohsuzu F. (2010). Associations between plasma C-reactive protein levels and the severities of coronary and aortic atherosclerosis. J. Atheroscler. Thromb..

[52] Lai M.M., Li C.I., Kardia S.L., Liu C.S., Lin W.Y., Lee Y.D., Chang P.C., Lin C.C., Li T.C. (2010). Sex difference in the association of metabolic syndrome with high sensitivity C-reactive protein in a Taiwanese population. BMC Public Health.

[53] Wee C.C., Mukamal K.J., Huang A., Davis R.B., McCarthy E.P., Mittleman M.A. (2008). Obesity and C-reactive protein levels among white, black, and hispanic US adults. Obesity.

[54] Flores-Alfaro E., Fernández-Tilapa G., Salazar-Martínez E., Cruz M., Illades-Aguiar B., Parra-Rojas I. (2012). Common variants in the CRP gene are associated with serum C-reactive protein levels and body mass index in healthy individuals in Mexico. Genet. Mol. Res..

[55] Hanly J.G., O’Keeffe A.G., Su L., Urowitz M.B., Romero-Diaz J., Gordon C., Bae S.C., Bernatsky S., Clarke A.E., Wallace D.J. (2015). The frequency and outcome of lupus nephritis: Results from an international inception cohort study. Rheumatology.

[56] Hermansen M.L.F., Lindhardsen J., Torp-Pedersen C., Faurschou M., Jacobsen S. (2016). Incidence of systemic lupus erythematosus and lupus nephritis in Denmark: A nationwide cohort study. J. Rheumatol..

[57] Maningding E., Dall’Era M., Trupin L., Murphy L.B., Yazdany J. (2020). Racial and Ethnic Differences in the Prevalence and Time to Onset of Manifestations of Systemic Lupus Erythematosus: The California Lupus Surveillance Project. Arthritis Care Res..

[58] Wells D.K., Ward M.M. (2010). Nephritis and the risk of acute myocardial infarction in patients with systemic lupus erythematosus. Clin. Exp. Rheumatol..

[59] Formiga F., Meco J.F., Pinto X., Jacob J., Moga I., Pujol R. (2001). Lipid and lipoprotein levels in premenopausal systemic lupus erythematosus patients. Lupus.

[60] Chaabane S., Messedi M., Akrout R., Ben Hamad M., Turki M., Marzouk S., Keskes L., Bahloul Z., Rebai A., Ayedi F. (2018). Association of hyperhomocysteinemia with genetic variants in key enzymes of homocysteine metabolism and methotrexate toxicity in rheumatoid arthritis patients. Inflamm. Res..

[61] Oosterom N., de Jonge R., Smith D.E.C., Pieters R., Tissing W.J.E., Fiocco M., van Zelst B.D., van den Heuvel-Eibrink M.M., Heil S.G. (2019). Changes in intracellular folate metabolism during high-dose methotrexate and Leucovorin rescue therapy in children with acute lymphoblastic leukemia. Tiziani S, editor. PLoS ONE.

[62] Costedoat-Chalumeau N., Dunogué B., Morel N., Le Guern V., Guettrot-Imbert G. (2014). Hydroxychloroquine: A multifaceted treatment in lupus. Presse. Med..

[63] Tao C.Y., Shang J., Chen T., Yu D., Jiang Y.M., Liu D., Cheng G.Y., Xiao J., Zhao Z.Z. (2019). Impact of antimalarial (AM) on serum lipids in systemic lupus erythematosus (SLE) patients: A systematic review and meta-analysis. Medicine.

[64] Floris A., Piga M., Mangoni A.A., Bortoluzzi A., Erre G.L., Cauli A. (2018). Protective Effects of Hydroxychloroquine against Accelerated Atherosclerosis in Systemic Lupus Erythematosus. Mediat. Inflamm..

[65] Pagler T.A., Neuhofer A., Laggner H., Strobl W., Stangl H. (2007). Cholesterol efflux via HDL resecretion occurs when cholesterol transport out of the lysosome is impaired. J. Lipid Res..

[66] Ignatescu M.C., Kletzmayr J., Födinger M., Bieglmayer C., Hörl W.H., Sunder-Plassmann G. (2002). Influence of mycophenolic acid and tacrolimus on homocysteine metabolism. Kidney Int..

[67] Segal R., Baumoehl Y., Elkayam O., Levartovsky D., Litinsky I., Paran D., Wigler I., Habot B., Leibovitz A., Sela B.A. (2004). anemia, serum vitamin B12, and folic acid in patients with rheumatoid arthritis, psoriatic arthritis, and systemic lupus erythematosus. Rheumatol. Int..

[68] Hunter P.M., Hegele R.A. (2017). Functional foods and dietary supplements for the management of dyslipidaemia. Nat. Rev. En-docrinol..

[69] Babiker R., Elmusharaf K., Keogh M.B., Saeed A.M. (2018). Effect of Gum Arabic (Acacia Senegal) supplementation on visceral adiposity index (VAI) and blood pressure in patients with type 2 diabetes mellitus as indicators of cardiovascular disease (CVD): A randomized and placebo-controlled clinical trial. Lipids Health Dis..

[70] Minami Y., Sasaki T., Arai Y., Kurisu Y., Hisamichi S. (2003). Diet and Systemic Lupus Erythematosus: A 4 Year Prospective Study of Japanese Patients. J. Rheumatol..

[71] Selhub J., Morris M.S., Jacques P.F. (2007). In vitamin B _12_ deficiency, higher serum folate is associated with increased total homocysteine and methylmalonic acid concentrations. Proc. Natl. Acad. Sci. USA.

[72] Chandrasekar B., Troyer D.A., Venkatraman J.T., Fernandes G. (1994). Dietary Omega-3 Lipids Delay the Onset and Progression of Autoimmune Lupus Nephritis by Inhibiting Transforming Growth Factor mRNA and Protein Expression. J. Autoimmun..

[73] Kinoshita K., Yoo B.S., Nozaki Y., Sugiyama M., Ikoma S., Ohno M., Funauchi M., Kanamaru A. (2003). Retinoic Acid Reduces Autoimmune Renal Injury and Increases Survival in NZB/W F 1 Mice. J. Immunol..

[74] Weimann J., Weiser H. (1992). Effects of Antioxidant Vitamins C, E, and p-Carotene on Immune Functions in MRL/lpr Mice and Rats. Ann. N. Y. Acad. Sci..

[75] Strickland F.M., Hewagama A., Wu A., Sawalha A.H., Delaney C., Hoeltzel M.F., Yung R., Johnson K., Mickelson B., Richardson B.C. (2013). Diet Influences Expression of Autoimmune-Associated Genes and Disease Severity by Epigenetic Mechanisms in a Transgenic Mouse Model of Lupus: Diet, DNA Methylation, and Lupus. Arthritis Rheum..

[76] Piantoni S., Andreoli L., Scarsi M., Zanola A., Dall’Ara F., Pizzorni C., Cutolo M., Airò P., Tincani A. (2015). Phenotype modifications of T-cells and their shift toward a Th2 response in patients with systemic lupus erythematosus supplemented with different monthly regimens of vitamin D. Lupus.

[77] Soni C., Sinha I., Fasnacht M.J., Olsen N.J., Rahman Z.S.M., Sinha R. (2019). Selenium supplementation suppresses immunological and serological features of lupus in B6. Sle1b mice. Autoimmunity.

[78] Brown A.C. (2000). Lupus erythematosus and nutrition: A review of the literature. J. Ren. Nutr..

[79] Perl A., Hanczko R., Lai Z.W., Oaks Z., Kelly R., Borsuk R., Asara J.M., Phillips P.E. (2015). Comprehensive metabolome analyses reveal N-acetylcysteine-responsive accumulation of kynurenine in systemic lupus erythematosus: Implications for activation of the mechanistic target of rapamycin. Metabolomics.

[80] Lai Z.W., Hanczko R., Bonilla E., Caza T.N., Clair B., Bartos A., Miklossy G., Jimah J., Doherty E., Tily H. (2012). *N* -acetylcysteine reduces disease activity by blocking mammalian target of rapamycin in T cells from systemic lupus erythematosus patients: A randomized, double-blind, placebo-controlled trial. Arthritis Rheum..

[81] Procaccini C., De Rosa V., Galgani M., Carbone F., Cassano S., Greco D., Qian K., Auvinen P., Calì G., Stallone G. (2012). Leptin-Induced mTOR Activation Defines a Specific Molecular and Transcriptional Signature Controlling CD4 ^+^ Effector T Cell Responses. J. Immunol..

[82] Leiba A., Amital H., Gershwin M.E., Shoenfeld Y. (2001). Diet and lupus. Lupus.

[83] McMahon M., Hahn B.H., Skaggs B.J. (2011). Systemic lupus erythematosus and cardiovascular disease: Prediction and potential for therapeutic intervention. Expert Rev. Clin. Immunol..

[84] Pestka J.J. (2010). n-3 Polyunsaturated fatty acids and autoimmune-mediated glomerulonephritis. Prostaglandins Leukot. Essent. Fatty Acids..

[85] Meyer B.J., Mann N.J., Lewis J.L., Milligan G.C., Sinclair A.J., Howe P.R.C. (2003). Dietary intakes and food sources of omega-6 and omega-3 polyunsaturated fatty acids. Lipids.

[86] Duffy E.M., Meenagh G.K., McMILLAN S.A., Strain J.J., Hannigan B.M., Bell A.L. (2004). The Clinical Effect of Dietary Supplementation with Omega-3 Fish Oils and/or Copper in Systemic Lupus Erythematosus. J. Rheumatol..

[87] Petri M. (2001). Diet and systemic lupus erythematosus: From mouse and monkey to woman?. Lupus.

[88] Fassett R.G., Gobe G.C., Peake J.M., Coombes J.S. (2010). Omega-3 Polyunsaturated Fatty Acids in the Treatment of Kidney Disease. Am. J. Kidney Dis..

[89] Halade G.V., Rahman M.M., Bhattacharya A., Barnes J.L., Chandrasekar B., Fernandes G. (2010). Docosahexaenoic Acid-Enriched Fish Oil Attenuates Kidney Disease and Prolongs Median and Maximal Life Span of Autoimmune Lupus-Prone Mice. J. Immunol..

[90] Carracedo M., Artiach G., Arnardottir H., Bäck M. (2019). The resolution of inflammation through omega-3 fatty acids in atherosclerosis, intimal hyperplasia, and vascular calcification. Semin. Immunopathol..

[91] Conte M.S., Desai T.A., Wu B., Schaller M., Werlin E. (2018). Pro-resolving lipid mediators in vascular disease. J. Clin. Invest..

[92] Liao X., Ren J., Wei C.H., Ross A.C., Cecere T.E., Jortner B.S., Ahmed S.A., Luo X.M. (2015). Paradoxical Effects of All-Trans-Retinoic Acid on Lupus-Like Disease in the MRL/lpr Mouse Model. PLoS ONE.

[93] De Lema G.P., Lucio-Cazaña F.J., Molina A.N.A., Luckow B., Schmid H., de Wit C., Moreno-Manzano V., Banas B., Mampaso F., Schlöndorff D. (2004). Retinoic acid treatment protects MRL/lpr lupus mice from the development of glomerular disease. Kidney Int..

[94] Patavino T., Brady D.M. (2001). Natural Medicine and Nutritional Therapy as an Alternative Treatment in Systemic Lupus Erythematosus. Altern. Med. Rev..

[95] Padayatty S.J., Katz A., Wang Y., Eck P., Kwon O., Lee J.H., Chen S., Corpe C., Dutta A., Dutta S.K. (2003). Vitamin C as an Antioxidant: Evaluation of Its Role in Disease Prevention. J. Am. Coll. Nutr..

[96] Maeshima E., Liang X.M., Goda M., Otani H., Mune M. (2007). The efficacy of vitamin E against oxidative damage and autoantibody production in systemic lupus erythematosus: A preliminary study. Clin. Rheumatol..

[97] Varghese B., Haase N., Low P.S. (2007). Depletion of Folate-Receptor-Positive Macrophages Leads to Alleviation of Symptoms and Prolonged Survival in Two Murine Models of Systemic Lupus Erythematosus. Mol. Pharm..

[98] Ardoin S., Sandborg C., Schanberg L. (2007). Review: Management of dyslipidemia in children and adolescents with systemic lupus erythematosus. Lupus.

[99] Bikle D.D. (2014). Vitamin D Metabolism, Mechanism of Action, and Clinical Applications. Chem. Biol..

[100] Dankers W., Colin E.M., van Hamburg J.P., Lubberts E. (2017). Vitamin D in Autoimmunity: Molecular Mechanisms and Therapeutic Potential. Front. Immunol..

[101] Illescas-Montes R., Melguizo-Rodríguez L., Ruiz C., Costela-Ruiz V.J. (2019). Vitamin D and autoimmune diseases. Life Sci..

[102] Penna G., Adorini L. (2000). 1α,25-Dihydroxyvitamin D 3 Inhibits Differentiation, Maturation, Activation, and Survival of Dendritic Cells Leading to Impaired Alloreactive T Cell Activation. J Immunol.

[103] Smolders J., Peelen E., Thewissen M., Cohen Tervaert J.W., Menheere P., Hupperts R., Damoiseaux J. (2010). Safety and T Cell Modulating Effects of High Dose Vitamin D3 Supplementation in Multiple Sclerosis. PLoS ONE.

[104] Lavi Arab F., Rastin M., Faraji F., Zamani Taghizadeh Rabe S., Tabasi N., Khazaee M., Haghmorad D., Mahmoudi M. (2015). Assessment of 1,25-dihydroxyvitamin D3 effects on Treg cells in a mouse model of systemic lupus erythematosus. Immunopharmacol. Immunotoxicol..

[105] Lemire J.M., Ince A., Takashima M. (1992). 1,25-Dihydroxyvitamin D 3 Attenuates of Expression of Experimental Murine Lupus of MRL/1 Mice. Autoimmunity.

[106] Reynolds J.A., Haque S., Williamson K., Ray D.W., Alexander M.Y., Bruce I.N. (2016). Vitamin D improves endothelial dysfunction and restores myeloid angiogenic cell function via reduced CXCL-10 expression in systemic lupus erythematosus. Sci. Rep..

[107] Manson J.E., Bassuk S.S., Lee I.M., Cook N.R., Albert M.A., Gordon D., Zaharris E., MacFadyen J.G., Danielson E., Lin J. (2012). The VITamin D and OmegA-3 TriaL (VITAL): Rationale and design of a large randomized controlled trial of vitamin D and marine omega-3 fatty acid supplements for the primary prevention of cancer and cardiovascular disease. Contemp. Clin. Trials..

[108] Jiao H., Acar G., Robinson G.A., Ciurtin C., Jury E.C., Kalea A.Z. (2022). Diet and Systemic Lupus Erythematosus (SLE): From Supplementation to Intervention. IJERPH.

[109] Albert C.M. (2009). Effect of Folic Acid and B-Vitamins on Risk of Cardiovascular Events and Total Mortality among Women at High Risk for Cardiovascular Disease: A Randomized Trial. JAMA.

[110] Lozovoy M.A.B., Simão A.N.C., Morimoto H.K., Scavuzzi B.M., Iriyoda T.V.M., Reiche E.M.V., Cecchini R., Dichi I. (2015). Fish oil n-3 fatty acids increase adiponectin and decrease leptin levels in patients with systemic lupus erythematosus. Marine Drugs..

[111] Wright S., O’Prey F.M., McHenry M.T., Leahey W.J., Devine A.B., Duffy E.M., Johnston D.G., Finch M.B., Bell A.L., McVeigh G.E. (2008). A randomised interventional trial of ω-3-polyunsaturated fatty acids on endothelial function and disease activity in systemic lupus erythematosus. Ann. Rheum. Dis..

[112] Gorczyca D., Szponar B., Paściak M., Czajkowska A., Szmyrka M. (2022). Serum levels of n-3 and n-6 polyunsaturated fatty acids in patients with systemic lupus erythematosus and their association with disease activity: A pilot study. Scand. J. Rheumatol..

[113] Tam L.S., Li E.K., Leung V.Y.F., Griffith J.F., Benzie I.F.F., Lim P.L., Whitney B., Lee V.W.Y., Lee K.K.C., Thomas G.N. (2005). Effects of vitamins C and E on oxidative stress markers and endothelial function in patients with systemic lupus erythematosus: A double blind, placebo controlled pilot study. J. Rheumatol..

[114] Vianna A.C.A., Mocelin A.J., Matsuo T., Morais-Filho D., Largura A., Delfino V.A., Soares A.E., Matni A.M. (2007). Uremic hyperhomocysteinemia: A randomized trial of folate treatment for the prevention of cardiovascular events. Hemodial. Int..

[115] Zhang P., Yang C., Guo H., Wang J., Lin S., Li H., Yang Y., Ling W. (2018). Treatment of coenzyme Q10 for 24 weeks improves lipid and glycemic profile in dyslipidemic individuals. J. Clin. Lipidol..

[116] Kießling G., Schneider J., Jahreis G. (2002). Long-term consumption of fermented dairy products over 6 months increases HDL cholesterol. Eur. J. Clin. Nutr..

[117] Gulati S., Misra A., Pandey R.M. (2017). Effects of 3 g of soluble fiber from oats on lipid levels of Asian Indians—A randomized controlled, parallel arm study. Lipids Health Dis..

[118] Mottaghi A., Ebrahimof S., Angoorani P., Saboor-Yaraghi A.A. (2014). Vitamin A Supplementation Reduces IL-17 and RORc Gene Expression in Atherosclerotic Patients. Scand. J. Immunol..

[119] Rashidi B.H., Mohammad Hosseinzadeh F., Alipoor E., Asghari S., Yekaninejad M.S., Hosseinzadeh-Attar M.J. (2020). Effects of Selenium Supplementation on Asymmetric Dimethylarginine and Cardiometabolic Risk Factors in Patients with Polycystic Ovary Syndrome. Biol. Trace Elem. Res..

[120] Nuttall S.L. (2003). Cardiovascular risk in systemic lupus erythematosus--evidence of increased oxidative stress and dyslipidaemia. Rheumatology.

[121] Wójcik P., Gęgotek A., Žarković N., Skrzydlewska E. (2021). Oxidative Stress and Lipid Mediators Modulate Immune Cell Functions in Autoimmune Diseases. IJMS.

[122] Hlais S., Reslan D.R.A., Sarieddine H.K., Nasreddine L., Taan G., Azar S., Obeid O.A. (2012). Effect of Lysine, Vitamin B6, and Carnitine Supplementation on the Lipid Profile of Male Patients With Hypertriglyceridemia: A 12-Week, Open-Label, Randomized, Placebo-Controlled Trial. Clin. Ther..

